# Tuberculosis arthritis of the sternoclavicular joint after uncomplicated *falciparum* malaria: a case report

**DOI:** 10.1186/s12941-017-0219-8

**Published:** 2017-06-05

**Authors:** Boundia Djiba, Baidy Sy Kane, Mamadou Alpha Diallo, Khadim Diongue, Ngoné Diaba Diack, Hamidou Deme, Mouhamed Dieng, Maimouna Sow, Daouda Ndiaye, Abdoulaye Pouye

**Affiliations:** 1 Service de Médecine Interne, Hôpital Aristide Le Dantec, Université Cheikh Anta Diop de Dakar, Avenue Cheikh Anta Diop, Fann, BP 5005, Dakar, Senegal; 2 Laboratoire de Parasitologie-Mycologie, Hôpital Aristide Le Dantec, Université Cheikh Anta Diop de Dakar, Avenue Cheikh Anta Diop, Fann, BP 5005, Dakar, Senegal; 3 Service d’Imagerie Medicale, Hôpital Aristide Le Dantec, Université Cheikh Anta Diop de Dakar, Avenue Cheikh Anta Diop, Fann, BP 5005, Dakar, Senegal

**Keywords:** Malaria, Tuberculosis, Arthritis, Immunity, Co-infection, Diagnosis

## Abstract

**Background:**

Malaria and tuberculosis are co-endemic in many developing countries. However their associations are rarely reported. Yet, it has been suggested that a pathological process may link the two diseases.

**Case presentation:**

A 20-year-old female patient was admitted in the internal medicine service of Aristide Le Dantec Hospital for uncomplicated malaria. She was previously treated for autoimmune hemolytic anaemia using prednisone at 5 mg per day. Clinical examination showed swelling in front of the sternoclavicular joint. She presented with fever and headache. Thick smear from blood revealed trophozoites of *P. falciparum* at parasite density of 52,300 parasites/μl. The Ziehl–Neelsen stained smear showed the presence of acid-fast bacilli from the fluid puncture of the swelling. *Mycobacterium tuberculosis* was further isolated in culture. The diagnosis of *falciparum* malaria co-infection with sternoclavicular tuberculosis was posed. The patient was treated successfully using antimalarial drugs subsequently followed by multidrug antitubercular therapy.

**Conclusion:**

Interactions between malaria and tuberculosis need to be largely and prospectively investigated and appropriate treatment should be undertaken.

## Background

Malaria and tuberculosis are two common infectious diseases in developing countries. About 3.8 billion are at risk of contracting malaria and the disease was responsible for more than 380,000 deaths in 2015 [1]. In the same year 10 million individuals contracted tuberculosis causing 1.8 million deaths [2]. Malaria and tuberculosis overlap geographically and co-infections should be common in the endemic regions [3]. However, their interactions remain poorly investigated. In this case, an acute sternoclavicular arthritis tuberculosis, which occurred after uncomplicated malaria, is described.

## Case presentation

A 20-year-old female student presented on the 12th of October 2016 to the Aristide Le Dantec Hospital. She presented with persistent fever and night sweats lasting for more than 1 year in a context of anorexia and weight loss. One week before she was hospitalized, it was noted a clinical picture of severe headache with exacerbation of the persistent fever accompanied by chills and profuse sweats.

**Fig. 1:**
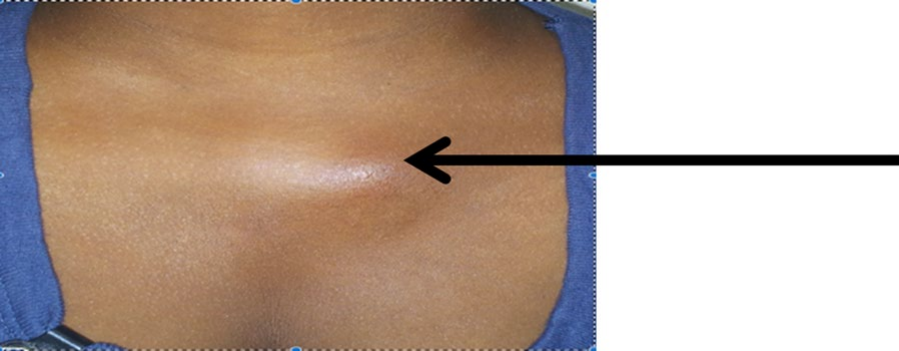
Swelling in the front of *left* sternoclavicular joint (*black arrow*)

**Fig. 2:**
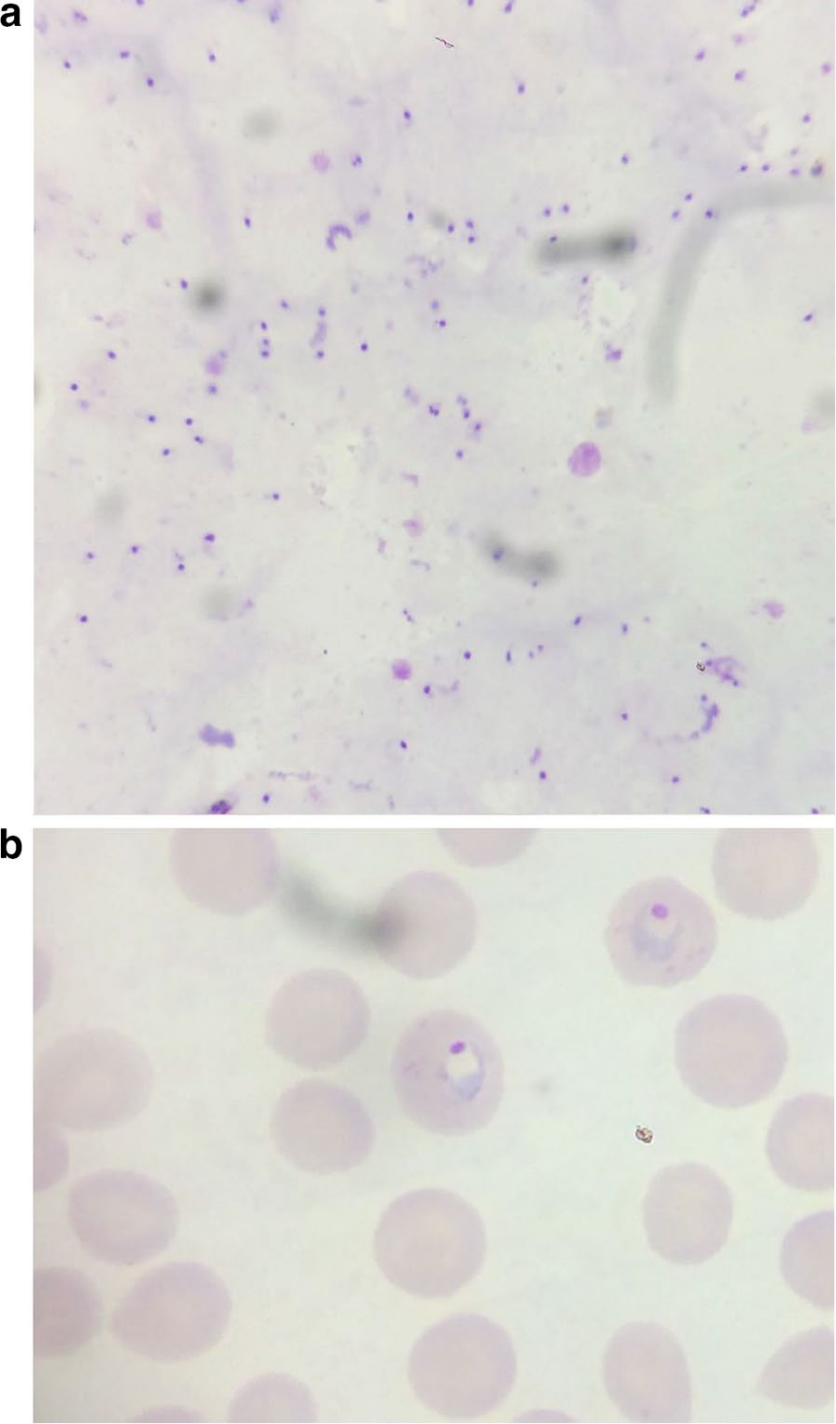
Giemsa-stained thick blood smear (**a**) and thin smear (**b**) showing trophozoite of *P. falciparum*

In her previous medical history, she was treated for autoimmune hemolytic anaemia by long-term corticosteroid therapy using prednisone at 5 mg per day. On examination the axillary temperature was 38.5 °C and blood pressure was 120/80 mmHg. Physical examination showed adenopathy measuring 2 cm on her left infraclavicular part (Fig. 1). The swelling was firm and painless, mobile and without fistulation. The rest of the examination was normal. Suspected malaria in the basis of clinical signs was confirmed by presence of trophozoite of *P. falciparum* on thick and thin smear with parasite density estimated at 52,300 parasites/μl (Fig. 2). The patient was then treated using artesunate/amodiaquine combination. She had her medical conditions improved 72 h after initial treatment but febricula still remained at 37.8 °C. Parasitaemia was cleared by day 3 after treatment.

Laboratory tests showed microcytic and hypochromic anaemia 6.1 g/dl haemoglobin (Normal: 12–16), thrombocytopaenia 72,000/mm^3^ (Normal: 150–400), ferritin > 2000 μg/l (Normal: 20–150), WBC 9400/mm^3^ (Normal: 4000–10,000). Biological inflammatory syndrome was observed as erythrocyte sedimentation rate (102 mm in the first hour) and C-reactive protein (96 mg/l) were elevated. Uraemia, creatininaemia and phosphorus and calcium were normal. HIV serological test was negative.

**Fig. 3:**
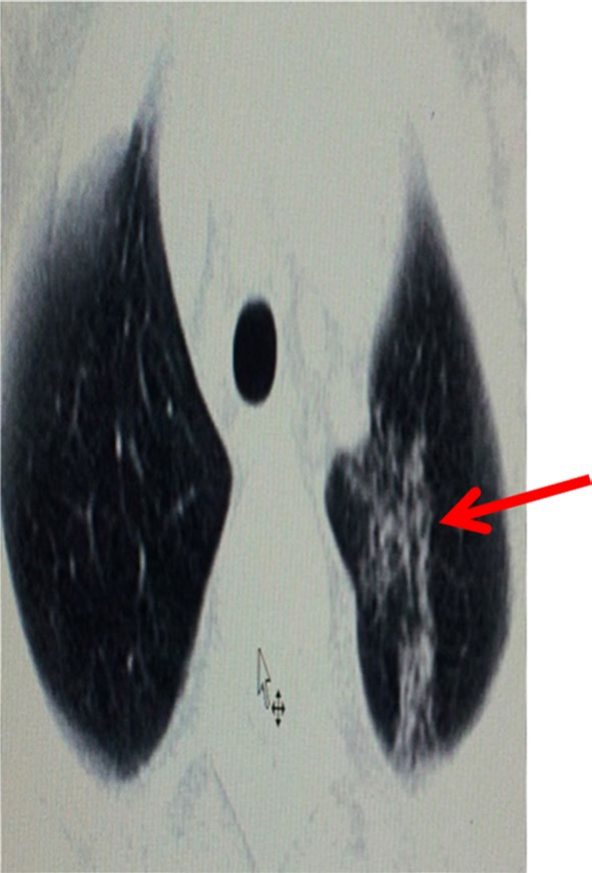
Chest CT scan showing small nodules on the apex making a tree-in-bud aspect (*red arrow*)

Chest X-ray showed reticular and nodular opacities disseminated throughout the lung fields (Fig. 3). The ultrasound of the sternoclavicular joint revealed regional fluid collection. Chest CT-scan showed small nodules in the parenchymal lungs windows resulting in tree-in-bud sign. Pre and retrosternal collections throughout the joint were observed in the mediastinal window. Bone window showed irregular bone surfaces on the sternoclavicular joint as a result of erosion process suggesting an arthritis.

Joint puncture collected purulent fluid. Microscopic examination after Ziehl–Nielsen staining of the fluid revealed acid-fast bacilli with count of 10 AFB per high-power field. Subsequently, *Mycobacterium tuberculosis* was isolated in Löwenstein–Jensen (LJ) solid medium where growth was detected after 3 weeks of incubation.

Tuberculosis arthritis of the sternoclavicular joint was decided and antitubercular therapy was initiated on November 15, 2016. Four first line antitubercular drugs treatment were given according to following schema: 2 months of rifampicin + isoniazid + ethambutol + pyrazinamide followed by 4 months of rifampicin + isoniazid.

**Fig. 4:**
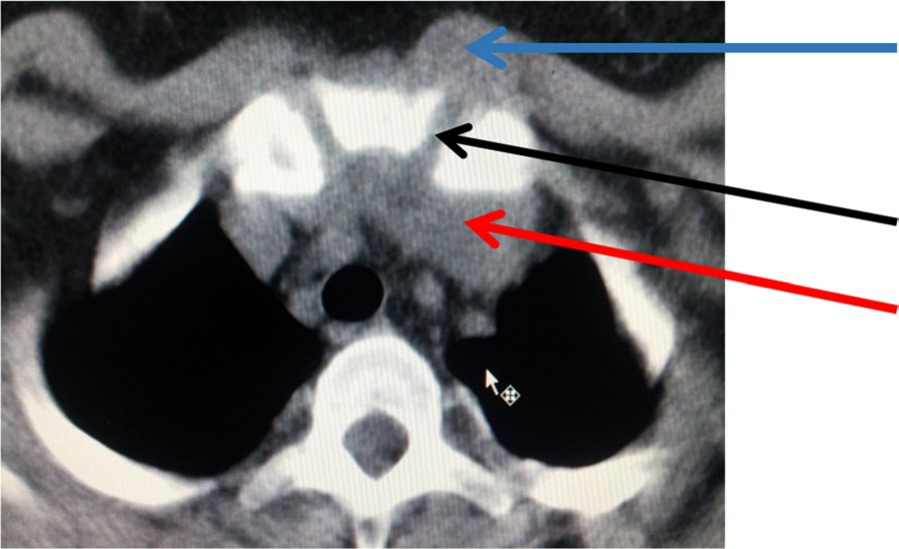
CT scan showing bone window with irregular and eroded bone surfaces of the sternoclavicular joint (*black arrow*). Bilateral fluid collection in pre-sternal (*blue arrow*) and retro-sternal (*red arrow*) are shown

After 2 months post therapy the patient’s symptoms completely disappeared with complete resolution of the lesion. CT scan showed complete healing of the lesion after 9 months of treatment (Fig. 4).

## Discussion

Given the considerable geographical overlap between *P. falciparum* and *M. tuberculosis*, it is highly probable that malaria–tuberculosis co-infections are common in endemic regions [2]. While the role of HIV in reactivating latent tuberculosis has been clearly established [4], little is known about malaria–tuberculosis interaction. Although acute malaria infections are known to be immunosuppressive [3], it is unclear how co-infections with malaria can affect the outcome of tuberculosis.

A prospective observational study suggests that malaria vector control measures could reduce significantly mortality among tuberculosis patients [5].

Previous studies suggested that malaria parasites could induce decrease of host’s effective humoral and cellular immune responses when challenged with *M. tuberculosis* infection [6, 7]. In vivo and in vitro experience models have shown that malaria parasites could modify host immunity and exacerbate mycobacterial infections [7–9]. Although their exact function remains unclear, CD4+ T cells have been suggested to play a key role in controlling both malaria and tuberculosis.

In this case, malaria was the primary diagnosis. Arthritis was guided by the presence of the swelling in the front of sternoclavicular joint. The presence of persistent fever associated with an acute type of arthritis (which is unusual in tuberculosis arthritis) was consistent with hypothesized sternoclavicular tuberculosis induced by uncomplicated *falciparum* malaria. In fact, in its usual course, sternoclavicular tuberculosis is described as a slowly progressive disease [10]. Also, it has been demonstrated that transient immunosuppression may be created during acute episode of malaria, which can lead to increased susceptibility to tuberculosis [3]. Early stage of malaria elicits the production of pro-inflammatory cytokines, IL-12 and IFN by Th1 T cells. Then, the response shifts to a predominantly Th2 response characterized by the production of IL-4 for complete clearance or resolution of infection [7]. Th2 is inhibitory to the production of IFN-gamma and IL-2 which are important to contain Mycobacterial growth [6]. Moreover, IL-10, a key cytokine in malaria immunity, is a negative regulator of Th1 responses. Thus, IL-10 antagonizes pro-inflammatory responses essential for protective immunity to *M. tuberculosis* [11].

Similarly, several other infectious diseases could have their outcome impacted during malaria co-infections. For instance, previous studies suggested that acute *P. falciparum* infection could impair EBV-specific T cell, allowing expansion of EBV infected B-cells resulting in Burkitt’s Lymphoma [12].

People living in malaria-endemic areas are frequently exposed to other pathogens mainly causing neglected infectious diseases, such as human African trypanosomiasis, leishmaniosis, schistosomiasis, etc. People living in those areas are more likely to present Th2 profile that could importantly affect the induction of an inflammatory Th2 type response needed to combat many pathogens [13].

Taken together, considerable attention need to be paid regarding interactions between malaria and other endemic infectious diseases.

## Conclusion

Both malaria and tuberculosis are endemic in tropical area and co-infections are probably common. The co-occurrence of both infections in individuals is becoming a new focus in tropical medicine.

Malaria could impair host immune response and lead to vulnerability to other infectious diseases such as tuberculosis. This case suggests that the role of malaria in tuberculosis reactivation is little considered as the two diseases are often seen separately. Therefore, a larger experimental study is needed to understand the actual interaction between malaria and tuberculosis and potentially to propose an inclusive strategy to fight both diseases.

## References

[1] World Health Organization (WHO) (2016). World malaria report.

[2] World Health Organization (WHO) (2016). Global actions and investments fall far short of those needed to end the global TB epidemic.

[3] Parra M, Derrick SC, Yang A, Tian J, Kolibab K, Oakley M, Perera LP, Jacobs WR, Kumar S, Morris SL (2011). Malaria infections do not compromise vaccine-induced immunity against tuberculosis in mice. PLoS ONE.

[4] Straetemans M, Bierrenbach AL, Nagelkerke N, Glaziou P, van der Werf MJ (2010). The effect of tuberculosis on mortality in HIV positive people: a meta-analysis. PLoS ONE.

[5] Colombatti R, Penazzato M, Bassani F, Vieira CS, Lourenço AA, Vieira F, Teso S, Giaquinto C, Riccardi F (2011). Malaria prevention reduces in-hospital mortality among severely ill tuberculosis patients: a three-step intervention in Bissau, Guinea-Bissau. BMC Infect Dis.

[6] Enwere GC, Ota MO, Obaro SK (1999). The host response in malaria and depression of defence against tuberculosis. Ann Trop Med Parasitol.

[7] Scott CP, Kumar N, Bishai WR, Manabe YC (2004). Short report: modulation of *Mycobacterium tuberculosis* infection by *Plasmodium* in the murine model. Am J Trop Med Hyg.

[8] Hawkes M, Li X, Crockett M, Diassiti A, Conrad Liles W, Liu J, Kain KC (2010). Malaria exacerbates experimental mycobacterial infection in vitro and in vivo. Microbes Infect.

[9] Mueller AK, Behrends J, Hagens K, Mahlo J, Schaible UE, Schneider BE (2012). Natural transmission of *Plasmodium berghei* exacerbates chronic tuberculosis in an experimental co-infection model. PLoS One.

[10] Kurtz B, Hauss PA, Druesne L, Chassagne P, Doucet J (2005). Tuberculosis arthritis of the sternoclavicular joint. Rev Med Interne.

[11] Blank J, Lars E, Behrends J, Jacobs T, Bianca ES (2016). One episode of self-resolving *Plasmodium yoelii* infection transiently exacerbates chronic *Mycobacterium tuberculosis* infection. Front Microbiol.

[12] Jayasooriya S, Hislop A, Peng Y, Croom-carter D, Jankey Y, Dong T, Rowland-jones S, Rickinson A, Walther M, Whittle H (2012). Revisiting the effect of acute *P. falciparum* malaria on Epstein-Barr virus : host balance in the setting of reduced malaria endemicity. PLoS One.

[13] Troye-Blomberg M, Berzins K (2008). Immune interactions in malaria co-infections with other endemic infectious diseases: implications for the development of improved disease interventions. Microbes Infect.

